# Langerhans cell histiocytosis with isolated meningeal involvement: findings on magnetic resonance imaging

**DOI:** 10.1590/0100-3984.2017.0034

**Published:** 2018

**Authors:** Bruno Niemeyer de Freitas Ribeiro, Bernardo Carvalho Muniz, Edson Marchiori

**Affiliations:** 1 Instituto Estadual do Cérebro Paulo Niemeyer - Departamento de Radiologia, Rio de Janeiro, RJ, Brazil.; 2 Universidade Federal do Rio de Janeiro (UFRJ), Rio de Janeiro, RJ, Brazil.

*Dear Editor,*

**Figure 1:**
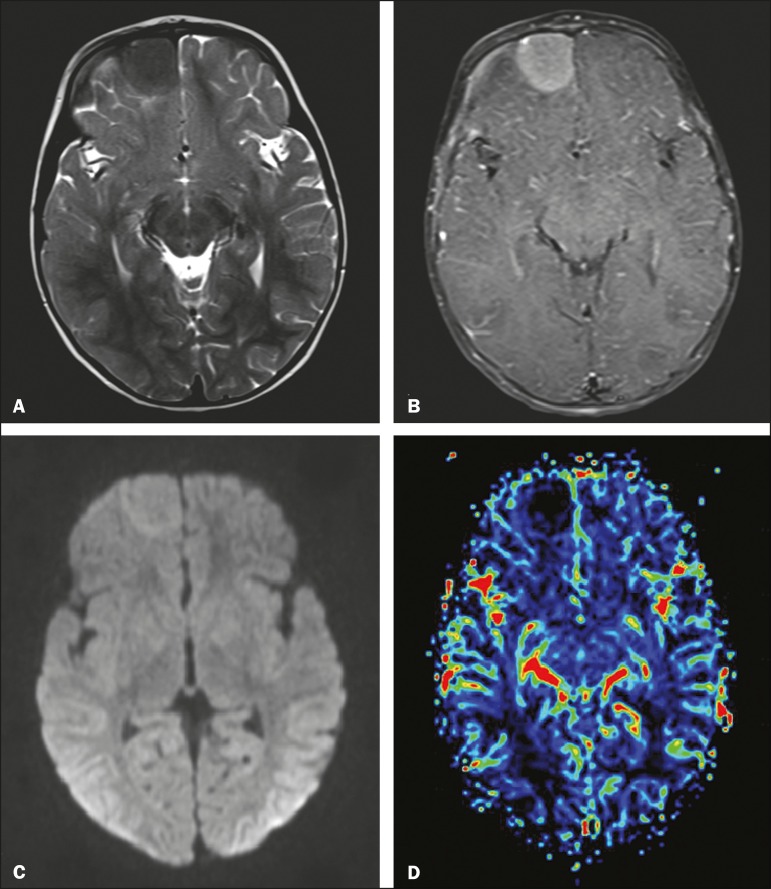
MRI. A: Axial T2-weighted image showing an extra-axial expansile lesion in the right frontal region, with precise, homogeneous and hypointense limits. B: Contrast-enhanced axial T1-weighted image showing marked gadolinium enhancement. C: Axial diffusion-weighted sequence showing a signal that is isointense in relation to that of the brain parenchyma. D: Map of the relative cerebral blood volume, demonstrating the absence of signs of hyperperfusion.

A 9-month-old male infant, with appropriate neuropsychomotor development, presented with an approximately two-week history of fever and seizures. The prenatal monitoring and delivery had been unremarkable. Serological tests for cytomegalovirus, toxoplasmosis, and HIV were negative, as was the venereal disease research laboratory test. The complete blood count, electrolyte analysis, and analysis of the cerebrospinal fluid all showed values within the normal ranges, with only a slight increase in erythrocyte sedimentation rate. Magnetic resonance imaging (MRI) of the brain showed an extra-axial expansile lesion exerting a compressive effect on the right frontal lobe, presenting hypointense signals in T1-weighted and T2-weighted sequences, together with marked gadolinium enhancement in T1-weighted sequence (Figure 1). Histopathological and immunohistochemical studies revealed granulomatous material with monoclonal Langerhans cells, Birbeck granules, and CD1a positivity, confirming the diagnosis of Langerhans cell histiocytosis (LCH).

LCH is a rare systemic disease of unknown cause, presenting a variable clinical course, from spontaneous regression and chronic recurrence to rapidly progressive deterioration with evolution to death^(1-4)^. It can occur at any age but is most common in children, primarily in those between 1 and 4 years of age, with an incidence of 1 case per 200,000 children^(3,4)^. Histopathological and immunohistochemical analysis reveals granulomatous infiltrates composed of monoclonal Langerhans cells, T cells, and eosinophils, with Birbeck granules and CD1a positivity^(1,5)^.

The main organs affected by LCH, in descending order of frequency, are the bones (in 80%), skin (in 33%), pituitary gland (in 25%), liver (in 15%), spleen (in 15%), and lung (in 5-10%); involvement of the pituitary gland typically manifests as diabetes insipidus^(5)^. In approximately 2-4% of the cases, there is involvement of the meninges, choroid plexus, pineal gland, and cerebral parenchyma, potentially provoking symptoms related to a compressive effect or cerebellopontine dysfunction, as well as neurodegenerative symptoms^(1,2,4,5)^.

Extra-axial involvement is more common in LCH because of the extent of the bone lesions affecting the skull, and the exclusive involvement of the meninges is rare, as demonstrated in this case^(1,2)^. MRI shows an expansile lesion, with a broad dural base, that is homogeneous, with a hypointense signal in T1-weighted sequences and an intermediate to hypointense signal in T2-weighted sequences, with moderate to marked gadolinium enhancement^(1,2,4)^.

There have been few reports of the behavior of histiocytosis in advanced MRI sequences. In our case, the lesion presented low signal intensity in a diffusion-weighted sequence, which is in accordance with the findings of Miyake et al.^(6)^, possibly secondary to the low cell content of the lesion. Classically, histiocytosis lesions do not show signs of hyperperfusion, because they are essentially lymphoproliferative disorders without neoangiogenesis. However, Hingwalaa et al.^(7)^ reported a case in which there was increased perfusion, with high positivity for CD34 and CD31, which are intrinsic markers of vascularization^(1,7)^. In the case reported here, there were no signs of increased perfusion.

The main differential diagnoses of LCH are forms of non-Langerhans histiocytosis (Rosai-Dorfman disease, Erdheim-Chester disease, and hemophagocytic syndrome), sarcoidosis, tuberculosis, meningioma, hemangiopericytoma, and solitary fibrous tumor^(1)^. Although there is no substantive consensus on the treatment of LCH, it is based on the location and number of lesions, the main therapeutic options being surgery and chemotherapy with various combinations of interferon, vinblastine, cladribine, and methotrexate.

Although uncommon, LCH should be considered in the differential diagnosis of extra-axial expansile lesions in children. It should be considered especially for lesions presenting an intermediate to hypointense signal in T2-weighted MRI sequences.
